# SDVL: Efficient and Accurate Semi-Direct Visual Localization

**DOI:** 10.3390/s19020302

**Published:** 2019-01-14

**Authors:** Eduardo Perdices, José María Cañas

**Affiliations:** RoboticsLab-URJC, Universidad Rey Juan Carlos, Fuenlabrada, 28943 Madrid, Spain; eperdices@gsyc.urjc.es

**Keywords:** Monocular Vision, SLAM

## Abstract

Visual Simultaneous Localization and Mapping (SLAM) approaches have achieved a major breakthrough in recent years. This paper presents a new monocular visual odometry algorithm able to localize in 3D a robot or a camera inside an unknown environment in real time, even on slow processors such as those used in unmanned aerial vehicles (UAVs) or cell phones. The so-called semi-direct visual localization (SDVL) approach is focused on localization accuracy and uses semi-direct methods to increase feature-matching efficiency. It uses inverse-depth 3D point parameterization. The tracking thread includes a motion model, direct image alignment, and optimized feature matching. Additionally, an outlier rejection mechanism (ORM) has been implemented to rule out misplaced features, improving accuracy especially in partially dynamic environments. A relocalization module is also included but keeping the real-time operation. The mapping thread performs an automatic map initialization with homography, a sampled integration of new points and a selective map optimization. The proposed algorithm was experimentally tested with international datasets and compared to state-of-the-art algorithms.

## 1. Introduction

Cameras are widely used sensors. Tablets, mobile phones, portable computers, and advanced robots are typically equipped with cameras. They provide images from the environment, and computer vision helps to extract useful information from them. Beyond object detection and tracking inside the images, the 3D position of the mobile camera itself can be extracted in real time using visual localization algorithms.

Two useful applications of visual localization are Mixed Reality and Augmented Reality in smartphones and tablets. For instance, the Tango project from Google, the IKEA Place app developed with Apple, or the inviZimals game from PlayStation are interesting examples. Visual localization can be performed there using fiducial markers (like Aruco, AprigTags or ARToolkit), but most advanced algorithms do not require any environment adaptation or artificial markers because modifying the environment adding markers may be not acceptable in many real settings. Recently, two software development kits for Augmented Reality in smartphones and tablets have been delivered: ARkit from Apple and ARcore from Google. They both include sophisticated visual localization algorithms.

In addition, visual localization is very useful in robotics, as the robots may take behavior decisions according to their position in the world. Autonomous cars in highways, humanoid robots in the RoboCup competition, drones for 3D path following and even robotic vacuum cleaners take advantage of knowing their position. For instance, the basic models of Roomba vacuum cleaner perform a random navigation algorithm because their odometry is mainly based on encoders. Odometry accumulates errors and does not provide a reliable absolute position estimation. Nevertheless, high-end models are equipped with cameras and visual localization algorithms which provide a better position estimation and this allows smarter coverage navigation algorithms and so, to clean faster and more systematically.

Initial visual localization algorithms such as CONDENSATION [1] require previous knowledge of the scene around the robot, possibly in terms of a map or in terms of a collection of beacons. Others calculate the 3D motion of a camera in real time without prior information of the environment, building a map of the scenario at the same time. They are known as visual SLAM (Simultaneous Localization and Mapping) algorithms and have been one of the main challenges in computer vision since the early 2000s. Their quality can be measured by looking at how they meet three requirements: accuracy, efficiency, and robustness.

Accuracy shows how close the estimation is from the ground truth and it can be measured as a root-mean-square error or as the probability of having an error below a certain threshold. The acceptable range greatly depends on the nature of the application. Efficiency refers to the speed of the estimation algorithm itself, whether it allows real-time operation or not. Greedy algorithms prevent their use in platforms with limited computing power. Robustness is the capability of delivering good position estimations despite many difficulties such as occlusions, low texture, fast movements, perception failures, mobile objects, etc. It may include relocalization capabilities, which refer to estimating the correct position again after the system gets completely lost, for instance after covering the camera and uncovering it in a new position of the same scenario. Another interesting concept is loop closure, which refers to the errors in the estimation when the camera returns to a previously visited place. The 3D estimations should be the same, but this is usually not the case as the algorithms use to accumulate errors.

One of the first visual SLAM approaches is the influential MonoSLAM (Monocular SLAM) [2,3]. It was based on an iterative Extended Kalman Filter that updates camera pose and map elements (3D points) on each iteration. It allowed real-time operation and tracked a limited the number of points. Subsequently, optimization methods such as PTAM (Parallel Tracking and Mapping) [4] proved to be more accurate and efficient than filtering approaches. They succesfully handle thousands of points [5], which provides robustness to the estimation. The main contribution of this new approach was to split up camera tracking and mapping into two separate threads. Only the tracking thread is required to work in real time. Most visual SLAM algorithms follow this structure, keep one thread for tracking and a separate one for mapping, and focus themselves on improving accuracy and efficiency.

Visual SLAM algorithms can be divided into two types according to how they extract information from images: direct or indirect methods. On the one hand indirect methods, also known as feature-based ones, extract features from images and match them in consecutive frames. Features are pixels with high intensity contrast and appear in areas of the image with texture. Typically, their 3D position and camera motion are estimated minimizing the reprojection error of the corresponding 3D points. PTAM follows this approach. On the other hand, direct methods use intensity values in the whole image and minimizes the photometric error to calculate camera motion. Usually these methods generate denser maps than feature-based methods but are computationally less efficient.

Dense maps help to achieve low localization errors and to work in real time with them many visual SLAM algorithms require the use of fast processors or even GPUs. However, these algorithms do not behave properly within devices with fewer computation capabilities, such as cell phones or unmanned aerial vehicles (UAVs). The approach presented in this paper is specifically designed to be used in slow processors, while being robust and accurate. To do so, a hybrid (both direct and indirect) algorithm, named semi-direct visual localization (SDVL), was designed and developed. An outlier rejection mechanism (ORM) is also explained to improve accuracy in semi-dynamic environments without risking accuracy.

The Section 2 briefly describes related works and state-of-the-art approaches. The Section 3 presents the SDVL design and point parameterization, while the following two sections detail its tracking and mapping modules. The Section 6 reviews the experiments, analysis and comparison performed to validate the approach. Our conclusions complete the paper.

## 2. Related Works

The density of the generated map is an important factor to improve the localization quality. While monoSLAM manages tens or hundreds of points as much, PTAM [4] was the first visual SLAM algorithm able to handle thousands of 3D points in real time. It extracts features from images using FAST corner detector algorithm [6] and matches these features in different frames to obtain their 3D position. Another PTAM contribution was a method to efficiently generate and store a 3D map, saving only some important frames (*keyframes*) in memory. Nevertheless, the proposed system was relatively simple and unable to manage large-scale maps.

Following the main PTAM architecture, [7] proposed an algorithm that detects loop closures and can successfully handle large maps. In addition, ORB-SLAM [8] improved accuracy replacing FAST by the ORB descriptors [9] for feature detection. However, all these approaches require fast computers to operate in real time.

Direct methods were first applied to visual SLAM by [10]. They aim to dense maps instead of the sparse maps created by previous feature-based SLAM approaches, but usually they require GPUs to work in real time. Other authors improved efficiency and accuracy using direct methods in following years: Dense Tracking and Mapping (DTAM) [11], Large-Scale Direct Monocular SLAM (LSD-SLAM) [12] and in particular Direct Sparse Odometry (DSO) [13,14], which achieved real-time tracking by reducing map density.

Furthermore, [15] proposed an efficient hybrid method, named Semi-direct Visual Odometry (SVO), capable of running at double real-time speed in slow processors. The main drawback of this algorithm is that it was designed to be used with down-looking cameras, common in drones, but it loses camera tracking easily in other settings.

More recently, the work of [16] improved visual SLAM accuracy in dynamic scenes detecting moving objects using deep learning and multi-view geometry.

Most of the mentioned works use RGB cameras, many times monocular ones. Other visual SLAM algorithms use RGB-D cameras, like RTABmap [17,18], and so they can take advantage of the depth information to solve the typical scale ambiguity when using monocular color cameras.

## 3. System Overview

The SDVL algorithm has been designed to be used in modest processors and must therefore be highly efficient. However, efficiency must not be detrimental to accuracy and robustness.

**Figure 1:**
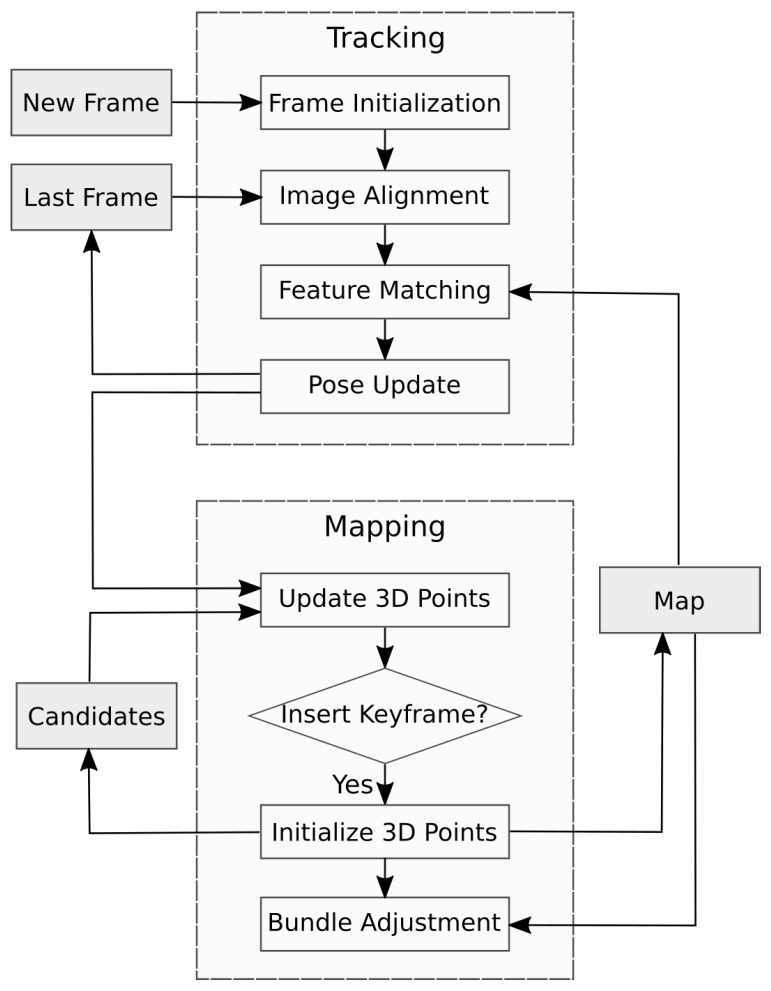
Tracking and mapping threads.

Following most of the SLAM algorithms structure (see Figure 1), SDVL has two separated threads: one estimates camera motion, whereas the other creates and refines a 3D map of the environment.

It uses a hybrid (direct and indirect) method to estimate camera motion, which will be explained in Section 4. In essence, it makes an initial approximation using direct methods and refines camera position by matching features, improving efficiency compared to classic feature-based methods.

The created map stores a set of 3D points using inverse-depth parameterization, as explained in Section 3.1. These 3D points are related to representative frames (keyframes) where they were observed. Furthermore, keyframes are connected to other keyframes sharing their field of view, that is, with several 3D points in common (at least 10), generating a graph of keyframes.

### 3.1. 3D Point Parameterization

Most SLAM approaches store 3D points directly as Euclidean coordinates (X,Y,Z). This parameterization simplifies points representation but has well-known disadvantages when using one single camera:

- New 3D points initially have an unknown depth until they are observed from two or more positions. SLAM approaches using Euclidean coordinates triangulate detected points from two [4] or more [8] keyframes to calculate their depth. This procedure generates outliers due to matching errors and causes a delay between points detection and point use.
- It is unable to properly handle distant points due to a low parallax between camera trajectory and points. It loses references that may be essential for camera tracking.

SDVL uses inverse-depth coordinates to represent 3D points. In particular, depth uncertainty is represented by a probability distribution as proposed in [19] and adapted to use inverse depth as in SVO [15].

Each 3D point is represented by the frame’s rotation $R_{F}$ and translation $T_{F}$ when the point was first detected, the reprojection ray $\left[v_{x},v_{y},v_{z}\right]$ in camera coordinates and the inverse depth $\rho =\frac{1}{Z}$, whose variance is $\sigma_{\rho }^{2}$. In contrast to Euclidean coordinates, inverse-depth parameterization can represent points in infinity when ρ=0.

Initially, both ρ and $\sigma_{\rho }^{2}$ are set to 1.0. Thus, with a 95% confidence region, point depth will be in range:

(1) $$\left[\frac{1}{\rho -2\sigma_{\rho }},\frac{1}{\rho +2\sigma_{\rho }}\right]$$

that is, using initial values and ignoring negative values, depth will be inside the interval $\left[\frac{1}{3},\infty \right]$.

**Figure 2:**
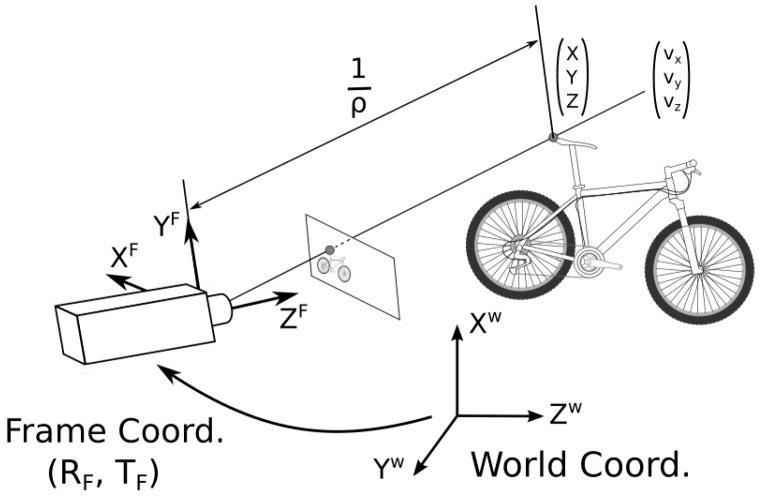
3D points parameterization and translation to Euclidean coordinates.

This parameterization can be translated to Euclidean coordinates as shown in Figure 2, using equation:

(2) $$\left(\begin{array}{c} X\\ Y\\ Z \end{array}\right)=R_{F}\frac{1}{\rho }\left(\begin{array}{c} v_{x}\\ v_{y}\\ v_{z} \end{array}\right)+T_{F}$$

In contrast to [15], SDVL uses this parameterization since each 3D point is created, instead of waiting until depth uncertainty is low. It prevents a delay between point detection and use, which may be indispensable when the camera is moving abruptly. In addition, distant points from camera position may have a great depth uncertainty but can be used to improve orientation accuracy.

Each point depth is updated with every frame until it converges, that is, until its depth uncertainty is small enough. To determine when a point fixes its 3D position, SDVL uses the linearity index *L* proposed by [20]:

(3) $$L=\frac{4\sigma_{\rho }}{\rho^{2}d}\left|cos\alpha \right|$$

where *d* is the distance between a 3D point and camera position and α is the parallax from each 3D point and the first and the last observation.

**Figure 3:**
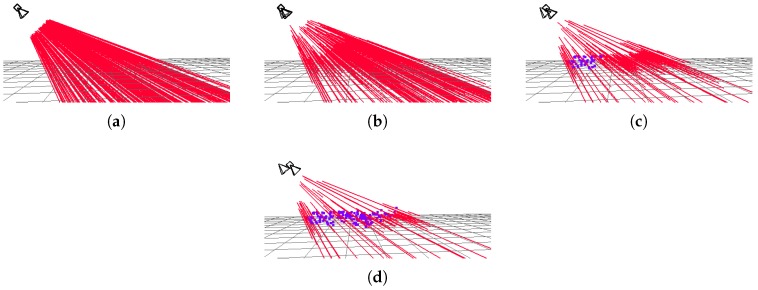
Depth convergence after 1 (**a**) 5 (**b**), 10 (**c**) and 15 (**d**) iterations. Red lines represent depth uncertainty in non-converged 3D points, blue points represent converged (and fixed) 3D points.

Thus, when the parallax is low *L* will be high, whereas when the parallax increases *L* is reduced. A point converges when *L* is lower than 0.1 (Figure 3).

## 4. Tracking

The tracking thread is responsible for calculating camera motion since the last iteration, based on camera images and 3D points stored in the map. In addition, camera tracking must be efficient to work in real time.

Once a new frame is obtained from the camera, the image is subsampled several times to obtain an image pyramid. Subsequently, features are extracted from each pyramid level using FAST algorithm. These features will serve to match 3D points in the current frame and (in certain cases) to create new 3D points.

The camera motion between previous ($x_{t-1}$) and current ($x_{t}$) pose is calculated in three steps.

### 4.1. Motion Model

Using a constant velocity model, current frame pose $x_{t_{i}}$ is initialized taking into account last frame pose $x_{t-1}$ and camera velocity in the last iteration.

### 4.2. Image Alignment

In this step, current frame ($F_{t}$) is aligned with previous frame ($F_{t-1}$) using direct methods, obtaining camera pose $x_{t_{e}}$. When these frames look at the same 3D point *p*, the intensity residual δF is defined as the photometric difference between the pixels where *p* is projected, taking into account the estimated depth of *p*.

Thus, $x_{t_{e}}$ is calculated minimizing δF for each 3D point $p_{i}$ detected in $F_{t-1}$:

(4) $$x_{t_{e}}=\underset{x_{t_{e},t-1}}{argmin}\frac{1}{2}\sum_{i}^{n}||\delta F\left(x_{t_{e},t-1},p_{i}\right){\left|\right|}^{2}$$

Residual intensity $\delta F(x_{t_{e},t-1},p_{i})$ is calculated using the *inverse compositional formulation* [21] using two 4 × 4 patches around $p_{i}$ projections in $F_{t}$ and $F_{t-1}$.

Frames will be aligned in each pyramid level, starting from high pyramid levels (with smaller images) and continuing with lower levels. This image alignment method was first proposed by [15], and makes it possible to efficiently perform alignment between two frames.

### 4.3. Feature Matching

Feature-based methods calculate camera pose through feature matching, using most of the tracking execution time. This is because each feature must be searched for in large image zones, just in case motion is abrupt and features have shifted greatly from the previous frame. In SDVL, image alignment obtains a camera pose $x_{t_{e}}$ close to real camera pose $x_{t}$, reducing search zones and improving efficiency.

Before performing feature matching, the image is divided into fixed-size cells. All 3D points visible in the previous frame are projected in the current frame and arranged in these cells according to their position in the image. No more than one point will be matched per cell; if the first point in each cell is not found, the algorithm will try to match another one until one matching is done or there are no more points remaining in that cell. Moreover, a maximum number of points is matched per iteration, so that computation time remains low.

**Figure 4:**
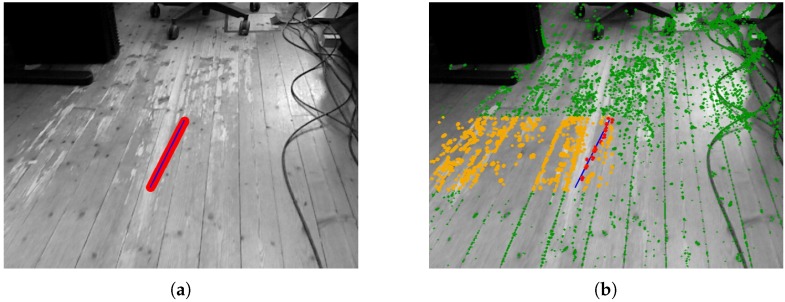
(**a**) Epipolar line corresponding to a 95% confidence region in depth (blue) and margin error around this line (red). (**b**) Fast point detected in image: skipped (green) ignored (orange) and used (red) during matching.

Depth uncertainty is taken into account when matching points. Thus, point search is performed in the epipolar line segment corresponding to a 95% confidence region in depth, with a small margin error around it (Figure 4a). Inside this search region, not all pixels are used, only those selected by FAST algorithm (Figure 4b).

There are two methods in SDVL to perform feature matching. The selected method has an influence on algorithm efficiency and accuracy, as will be shown in Section 6:

- Patch comparison: Small patches (8 pixels) are obtained around pixels when a 3D point is first observed and for each pixel selected in matching. Patches are compared using *Zero-Mean SSD* score [22] after warping features according to camera pose, similar to other feature-based algorithms [4].
- ORB descriptors: ORB descriptors are only extracted in pixels needed for matching, that is, the same pixels needed for patch comparison. Matching is performed computing the distance between two ORB descriptors, the same way as ORB-SLAM.

After properly matching *N* 3D points in the current frame, camera pose $x_{t}$ is calculated minimizing reprojection error according to equation:

(5) $$x_{t}=\underset{x_{t_{e}}}{argmin}\frac{1}{2}\sum_{i}^{n}\left|\right|p_{i}-f_{p}\left(x_{t_{e}},X_{i}\right){\left|\right|}^{2}$$

where $X_{i}$ is the 3D point position and $f_{p}\left(x_{t_{e}},X_{i}\right)$ is the projection function of $X_{i}$ in current $posex_{t_{e}}$.

### 4.4. Outlier Rejection Mechanism

The created map may contain 3D points located in a wrong position (outliers) in two situations:

- The point has not been properly triangulated, due mainly to matching issues. This situation is minimized in SDVL since points depth is calculated using several frames.
- The 3D point is part of a dynamic element and has changed its position since it was detected.

Most SLAM approaches can handle a reduced number of outliers, whenever the number of outliers is small compared to the total number of points used for camera tracking. However, if there are many outliers, accuracy may decrease, or localization may even be lost.

SDVL uses an ORM that takes place during feature tracking to rule out outliers before calculating current camera pose. This mechanism is based on [23], where a similar method for MonoSLAM-like approaches were proposed.

Thus, instead of using all matches found in the current frame, only a subset of 3D points is selected to calculate $x_{t}$. This subset is obtained iteratively using RANSAC [24]:

1. *m* matched 3D points are selected randomly, and camera pose is calculated minimizing reprojection error only with these points. By default, *m* is set to 5, since it is the minimal number of points needed to calculate the 3D displacement between two frames [25].
2. After updating camera position, the rest of matched 3D points are projected in $F_{t}$, saving points with a low reprojection error as inliers and the rest as outliers.
3. If the number of inliers in the current hypothesis is larger than in previous iterations, inliers and outliers are saved as the best hypothesis so far.

RANSAC algorithm iterates until the number of iterations performed is greater than the number of hypotheses $n_{h}$ which ensures that at least one hypothesis without outliers is selected with probability *p* (default 99%). This value is updated at each iteration according to equation:

(6) $$n_{h}=\frac{log(1-p)}{log(1-{\left(1-\epsilon \right)}^{m})}$$

where ϵ is the outlier ratio in the last iteration and *m* is the number of matched points randomly selected.

When RANSAC stops iterating, inliers from the best hypothesis are used to calculate $x_{t}$, ruling out outliers. Some of these outliers may correspond to high innovation inliers, that is, 3D points close to the camera that have shifted many pixels since the last iteration, so step 2 is repeated using $x_{t}$ to try to rescue some wrongly labeled outliers; if this occurs, $x_{t}$ is recalculated again taking into account these new inliers.

This procedure improves tracking reliability, as will be show in Section 6.

### 4.5. Relocalization

Matching ratio *r* is defined as matches made divided by matches attempted; *r* decreases when camera pose is inaccurate, and many 3D points are not found. Tracking is lost when *r* is lower than a threshold (0.2 by default) for several consecutive iterations. If this happens, the relocalization mechanism is activated.

This mechanism may not work in real time; consequently, some frames may be skipped without being analyzed. To relocalize the robot, keyframes stored in the map are compared iteratively to the current frame, beginning with the last keyframe until a valid keyframe is found. This procedure is performed in two steps:

1. The current image is aligned with the keyframe image using the same procedure as for tracking. The next step is only carried out if the average photometric error is low.
2. 3D points from keyframe are searched in the current image. If more than 20 points are correctly matched, the reprojection error between matches is minimized and the remaining keyframes are skipped.

Whenever a valid keyframe is found, tracking continues with the new estimated camera pose. On the other hand, if relocalization is not valid it will be performed again with the next frame received.

## 5. Mapping

The mapping thread manages the map of the environment surrounding the robot. The map is updated with each new frame and is used by the tracking thread to calculate camera pose.

Storing all frames and 3D points in the map is not feasible if the algorithm is to be efficient. The goal is to store only certain frames (keyframes) representing the scene without storing redundant information. Many SLAM approaches (like PTAM) insert only a few keyframes to maintain a low computational time, but this may lead the tracking to fail with strong camera movements. Therefore, SDVL follows ORB-SLAM [8] and DSO [13] policy, inserting many keyframes and deleting some of them afterwards to always keep large number of points in the current field of view. Keyframes are inserted when (1a) and (1b) conditions are met or when (2) condition is true:

- (1a): Enough iterations (at least 30) have passed since the last keyframe was saved.
- (1b): Number of 3D points seen in the last frame have decreased more than 10% since the last keyframe.
- (2): Number of 3D points seen in the last frame have decreased more than 30% since the last keyframe.

The first condition involves inserting a few keyframes when the camera is moving slowly, whereas the second condition ensures keyframe insertion in extreme camera motion. The number of keyframes is limited, so the map does not increase indefinitely; when this limit is reached, keyframes further away from the current camera position are deleted. All these threshold values were empirically selected by trial and error.

When a new keyframe is inserted in the map, the algorithm initializes new 3D points in areas not previously explored. These 3D points are saved in a list as *candidates* until they converge; these *candidates* are updated with each frame, even if they are not inserted as keyframes.

### 5.1. Map Initialization

To initialize the map, the algorithm needs two frames sharing their field of view. The camera displacement between both frames must be sufficiently large to retrieve the initial map properly.

The map initialization mechanism is automatic and needs no human interaction. It selects a large number of pixels (at least 50) from the first frame using FAST corner detector. These pixels are tracked in subsequent frames using Lucas-Kanade optical flow [26] until the average shift between tracked points is above a certain threshold. If many pixels are lost in this procedure, initialization is automatically restarted using the next frame obtained.

After choosing two frames and assuming that they observe a locally planar scene, the initial map is calculated as in [4]. An homography *H* is estimated from pixels shift using RANSAC [24] to minimize the reprojection error between each pair $\left(x_{i},y_{i}\right)$ and $\left(x_{i}^{\prime },y_{i}^{\prime }\right)$, corresponding to each pixel coordinates in both frames:

(7) $$\left(\begin{array}{c} x_{i}^{\prime }\\ y_{i}^{\prime }\\ 1 \end{array}\right)∼H\left(\begin{array}{c} x_{i}\\ y_{i}\\ 1 \end{array}\right)$$

Camera rotation and translation between both frames is obtained decomposing *H* and 3D points are calculated through triangulation.

### 5.2. New 3D Points Initialization

The map is continuously updated and extended as the camera moves around the environment, initializing new 3D points after inserting keyframes.

Only unexplored image zones are of interest to create new 3D points. The image is divided into cells and points already stored in the map are projected in the image to discard non-empty cells. One new 3D point is then created per empty cell. Each initialized 3D point belongs to the FAST feature with the highest Shi-Tomasi [27] score in each cell.

Initial depth from 3D points can be determined with a default value, such as mean depth from detected points during tracking, but if this value is unrealistic, matching may take longer in the following frames because uncertainty will be high. To avoid this situation, new points are searched in previous keyframes to set initial point depth through triangulation, using the matching technique explained in Section 4. This increases mapping time when points are created but reduces tracking time significantly in following iterations.

Point depth and uncertainty are now updated with each new frame until they converge, whenever they are matched in the current frame. If not found across many consecutive frames, they are deleted permanently. Moreover, when a point’s uncertainty decreases enough, its depth is fixed permanently.

### 5.3. Map Optimization

Keyframes poses and 3D points positions are optimized using Bundle Adjustment [28]. This algorithm has been widely used in feature-based SLAM approaches, because it significantly improves tracking accuracy.

Bundle Adjustment minimizes the reprojection error between *m* camera poses and *n* 3D points:

(8) $$min_{a_{j},b_{i}}\sum_{i=1}^{n}\sum_{j=1}^{m}v_{ij}d{\left(Q\left(a_{j},b_{i}\right),x_{ij}\right)}^{2}$$

where each camera is parameterized by a vector $a_{j}$ and each 3D point with a vector $b_{i}$. $Q(a_{j},b_{i})$ is the predicted projection of point *i* with camera position *j*, $x_{ij}$ the pixel where point *i* was detected, $d(p_{1},p_{2})$ the Euclidean distance between two pixels and $v_{ij}$ determines if point *i* was visible from position *j*.

SDVL uses Bundle Adjustment after map initialization and after inserting a new keyframe. To keep mapping time low, BA is only performed locally, using only keyframes sharing their field of view with the current keyframe.

In addition, only converged 3D points are used in BA, since this algorithm cannot handle depth uncertainty and using it to transform points to absolute coordinates prematurely may lead to inaccurate optimization.

## 6. Experimental Results

We used three popular datasets to evaluate the accuracy and efficiency of the proposed algorithm:

- TUM RGB-D: This dataset [29] contains 89 indoor sequences sorted in different categories. It provides different challenges, such as large sequences with loop closing, hard camera movements, robot kidnappings, dynamic elements, etc. Not all sequences are suitable for SLAM approaches, because some include pure rotations. To evaluate the algorithms, we used five sequences from this dataset: *Freiburg1 Floor* (F1F), *Freiburg2 Desk* (F2D), *Freiburg3 Walking Static* (F3W), *Freiburg2 360 Kidnap* (F2K) and *Freiburg1_xyz* (F1XYZ). The first two sequences are used to evaluate the algorithm in static environment, F3W contains dynamic elements, whereas F2K is suitable for testing the relocalization capability of SDVL after kidnapping. F1XYZ presents an indoor sequence around a desk with many translatory motions along the principal axes, it is good to test the behavior in high dynamism sequences.
- EUROC MAV: This dataset [30] uses a micro aerial vehicle (MAV) to record 11 sequences in 2 different scenarios. Sequences are labeled as easy, medium, and difficult according to camera speed and light conditions. In the experiments we used two sequences corresponding to the same scenario, *Machine Hall 01* (MH01) and *Machine Hall 02* (MH02). These sequences were recorded in a large industrial environment (400 m^2^) with a MAV starting and finishing at the same position.
- Zurich Urban MAV: This recent dataset [31] also uses a MAV to record one single sequence over 2 km in streets in Zurich (Switzerland), intended to evaluate visual odometry and SLAM approaches. In our experiments, we did not use the whole sequence to evaluate the algorithms, but only the first 9 thousand images corresponding to 5 min of recorded data (out of 81,169 images, 45 min), denoted in the experiments as ZU.

These sequences were selected to evaluate scenarios with different challenges: indoor and outdoor environments, large scenarios and small rooms, aerial vehicles, and handheld cameras, etc. Some of these datasets provide data from different sensors, including several cameras, GPS, or depth images. To perform the experiments only one camera was used, ignoring the other sensors.

SDVL results were compared with other known SLAM algorithms: PTAM [4], SVO [15], LSD-SLAM [12], ORB-SLAM [8] and DSO [13]. All these algorithms were run on an *Intel(R) Core(TM) i5-3330 CPU @ 3.00GHz* desktop computer with 8 GB RAM for comparison.

The results shown correspond to the most accurate execution after running each algorithm three times per sequence. Accuracy was compared using root-mean-square error (RMSE) as reference.

### 6.1. Accuracy Experiments

First, SDVL was analyzed comparing two versions of SDVL, using either patches or ORB descriptors for matching (SDVL-ORB). We use sequences MH01 and MH02 from EUROC MAV dataset to illustrate the differences between these configurations.

**Figure 5:**
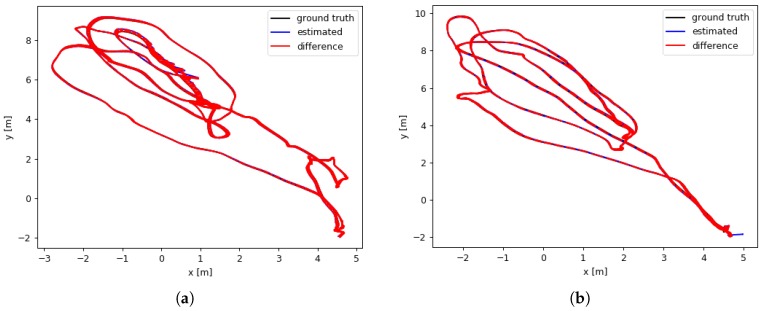
Difference between estimated pose and ground truth in MH01 (**a**) and MH02 (**b**) sequences using ORB descriptors for matching.

The first two rows of Table 1 show SDVL and SDVL-ORB accuracy in MH01 and MH02 sequences. Both configurations of the algorithm can complete both sequences with good accuracy. However, the ORB descriptors improve accuracy a little in both cases. The estimated and ground truth trajectory for these sequences using SDVL-ORB are shown in Figure 5.

### 6.2. Efficiency Experiments

Table 2 shows execution time (in milliseconds) itemized in tracking and mapping threads using two different computers, an ordinary computer desktop and an inexpensive ($100) Intel Compute Stick (STCK1A32WFC model). Most of the tracking time is spent initializing frames and matching features (including the ORM). Frame initialization consists of extracting FAST corners and calculating the image pyramid. Its execution time is quite stable and mainly depends on image size. Image alignment is very fast and provides a good initial estimation to feature matching. Matching is slower using ORB descriptors, since its extraction is about 3 times slower than with patches. Tracking thread works in real time both using patches and ORB descriptors in the desktop computer, which is mandatory for localization purposes. However only the first configuration can maintain real time in the Intel Compute Stick.

**Figure 6:**
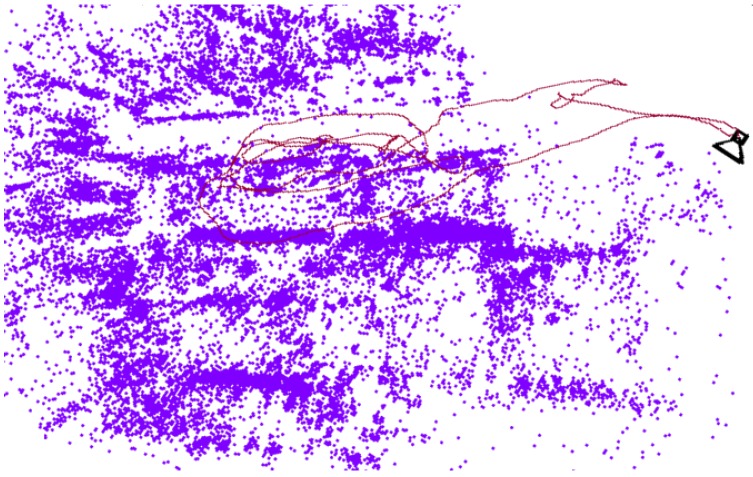
3D map created in sequence MH01 with SDVL-ORB.

On the other hand, the mapping thread is slower, especially when Bundle Adjustment optimization is performed, but this is not required to run in real time. Using ORB for matching features is faster in the mapping thread. This may initially seem surprising, but occurs because ORB descriptors are more discriminant than patches when selecting 3D points. This means that ORB selects better and fewer points for tracking, resulting in fewer points in the map to be updated, fewer points optimized with Bundle Adjustment, and ultimately faster mapping iterations. The map created with the first sequence using ORB descriptors is shown in Figure 6. It is not dense, which is similar to other feature-based methods.

### 6.3. Robustness Experiments

#### 6.3.1. Dynamic Environment

**Figure 7:**
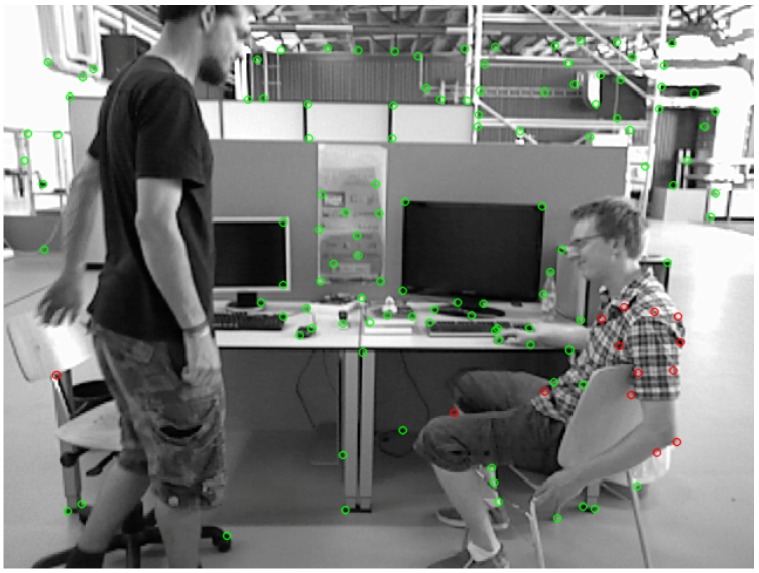
Screenshot obtained from sequence F3W. Points labeled as outliers are circled in red, whereas green circles represent inliers used to update camera pose.

**Figure 8:**
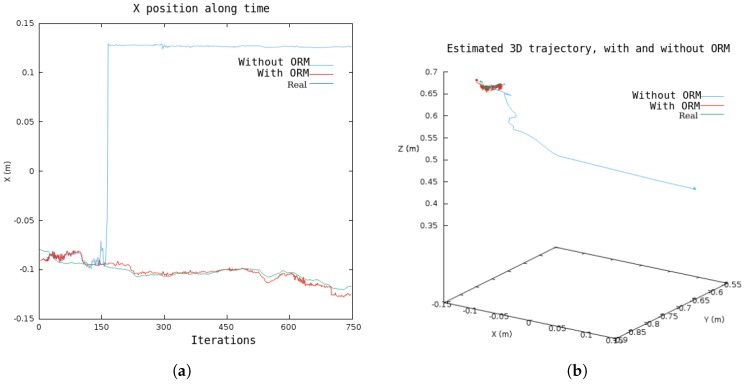
Estimated position and ground truth in sequence F3W with dynamic elements.

The ORM has been especially designed to improve accuracy in environments with dynamic elements and, hence, improve the robustness of the algorithm. The experiment performed to illustrate the importance of ORM uses sequence F3W from the TUM RGB-D dataset (Figure 7), where some elements of the scene (humans) stand up and walk in front of the camera, occupying most of the image. Figure 8 shows estimated and ground truth trajectories with and without ORM; without ORB the algorithm loses its 3D pose after a few seconds when a person crosses in front of the camera, whereas ORB only selects for tracking non-dynamic elements and keeps the camera pose stable.

#### 6.3.2. Kidnapping

This experiment shows SDVL capability to recover camera pose after losing it, using the relocalization mechanism. We use sequence F2K from the TUM RGB-D dataset, where a robot is moving around the environment and after a time the camera is occluded manually for a few seconds, simulating robot kidnapping. The robot is lost for a time until it closes the loop and returns close to its initial position.

**Figure 9:**
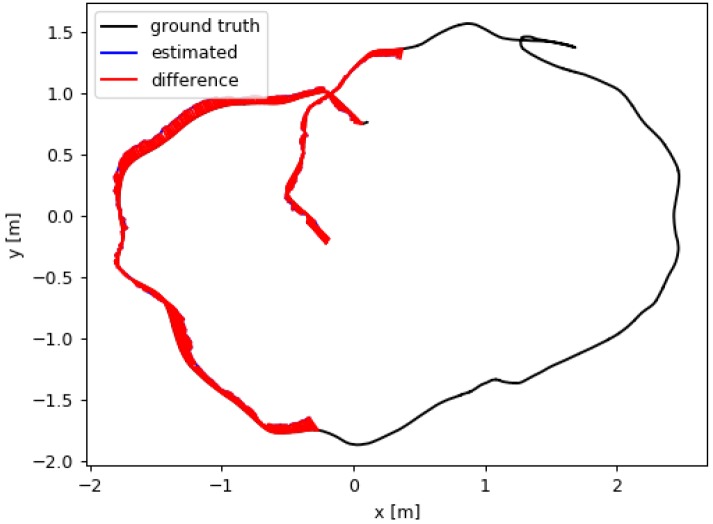
Kidnapping simulation using sequence F2K. Estimated and ground truth trajectories. Red line stops while camera pose is unknown due to kidnapping.

The third row of Table 1 shows the accuracy results for these experiments, while Figure 9 shows the estimated trajectory.

### 6.4. Empirical Comparison

The SDVL approach was compared to the most important state-of-the-art SLAM algorithms: PTAM [32], SVO [33], LSD-SLAM [34], ORB-SLAM [35] and DSO [36]. They all were downloaded from the original author software repositories. Loop closing features were deactivated in LSD-SLAM and ORB-SLAM to make a fair comparison. Furthermore, two configurations of the DSO algorithm were tested, a default one and a faster DSO alternative explained by the DSO authors. Two sequences from EUROC MAV and TUM RGB-D datasets and the first 5 min from Zurich Urban MAV dataset were used to perform the comparison.

Table 3 shows the accuracy results (in meters) obtained, and Table 4 shows the tracking time in milliseconds. Cells with an * mean that the algorithm has been able to complete partially the sequence before losing camera pose, while an X means that the algorithms lost the camera for pose most of the sequence or was not even able to initialize the camera pose.

ORB-SLAM and DSO (both standard and fast implementation) seem to be the most accurate algorithms. SVO is also highly accurate in some sequences but loses the camera pose in others, especially when fast camera motion is involved. PTAM and LSD-SLAM are less accurate in this comparative, the first frequently loses the camera pose and the second is the least accurate.

Table 4 shows the most efficient algorithm analyzed is SVO, with only a few milliseconds per iteration, whereas ORB-SLAM and DSO are the approaches with the longest execution time for tracking, being close to real-time operation. LSD-SLAM and DSO-Fast have intermediate times, whereas PTAM is not conclusive since it loses the camera pose in most of the experiments.

With the above results, some conclusions about robustness can be drawn. PTAM seems only to work in small spaces, whereas SVO also loses the camera pose when the camera spins fast, since its original implementation is focused on down-looking cameras. The rest of the algorithms are robust and can complete all the sequences.

As can be deduced from Table 4, most of the analyzed approaches perform tracking in real time in the computer desktop used for evaluation. However, as shown in Table 2, performing the same experiments in an Intel Compute Stick increases execution times by 300 to 400% compared to the computer desktop. This means that only these algorithms able to perform tracking up to 10 ms in Table 2 will be able to run in real time in these kinds of devices, which is the case of SDVL.

**Figure 10:**
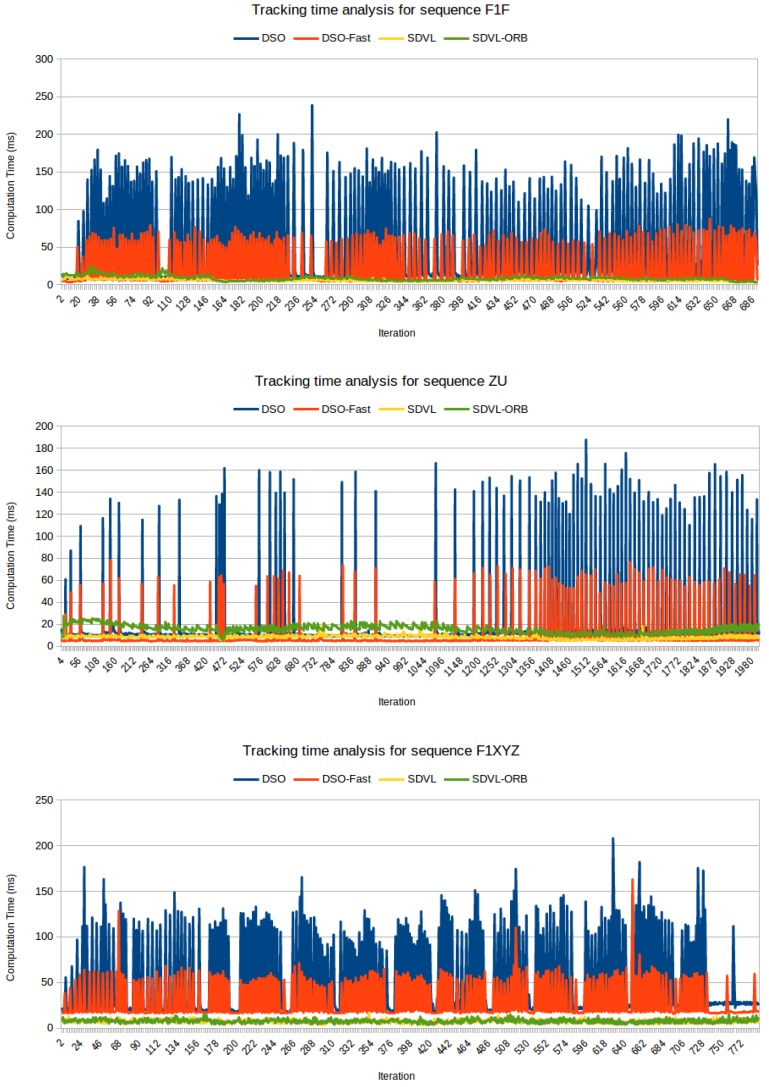
Tracking time evolution for F1F sequence, ZU sequence (first 2000 iterations) and F1XYZ sequence.

The DSO and DSO-fast computation times greatly depend on the dynamism of the sequence. In sequences with much movement and many new points, such as F1F in Table 4 (a camera moves fast over the floor) or F1XYZ in Figure 10, their computation times grow significantly, doubling their values in sequences with low dynamism, such as ZU in Table 4 (a drone moves very slowly always looking at a far point). As can be seen in Figure 10 the computation times of DSO and DSO-fast tracking present peaks whenever new points are incorporated. In addition, so, they have high typical deviations. In high dynamism scenarios (like F1F or F1XYZ) that happens frequently. In low dynamism scenarios (like ZU) there are few peaks and their efficiency is quite good. In contrast, the computation time of SDVL tracking is relatively stable, with low typical deviation even in scenarios with high dynamism.

This has been analyzed in detail in three sequences (F1F, ZU and F1XYZ), and shown in Figure 10. Iterations without peaks are from intervals when the camera was stable or there was no much movement. For the F1XYZ sequence (Figure 10) the average iteration times were 7.33 ms for SDVL, 7.66 ms for SDVL-ORB, 48.03 ms for DSO and 27.90 ms for DSO-fast. The corresponding typical deviations were 2.01 ms, 2.15 ms, 41.89 and 18.25 ms.

DSO and DSO-fast are very good algorithms, two times more accurate than the proposed SDVL, maybe because they spend two times more computation time, roughly speaking. SDVL is a good trade off solution for applications with limited computing power and where accuracy below 12 cm is suitable.

## 7. Conclusions

In this paper, a SDVL algorithm is proposed, focused on efficiency in modest processors. The designed hybrid method improves tracking efficiency using direct methods to estimate an initial camera pose before performing feature tracking, reducing search areas and execution time.

Regarding comparing the two matching methods proposed, ORB seems to provide more accuracy, while using patches ensures a faster tracking. The configuration selected will depend on localization purpose and computational capability.

The algorithm has an ORM which improves localization stability and accuracy in semi-dynamic environments, as shown in experiments.

Compared to other SLAM algorithms, both SDVL and SDVL-ORB keep a balance between accuracy and efficiency. Other algorithms such as ORB-SLAM and DSO are more accurate, but their tracking time is longer. On the other hand, SVO is more efficient but it is not robust enough to be used for general purposes. This makes SDVL suitable for use in devices with low computational capability, being accurate, efficient, and robust.

Regarding future research lines, the authors are working on building a tool to objectively measure the performance of SLAM algorithms, using international datasets and well accepted quality metrics for accuracy, efficiency, and robustness. A second on-going work is to extend the algorithm to include a loop closure module.

## Figures and Tables

**Table 1:** SDVL accuracy.

	SDVL		SDVL-ORB	
Sequence	RMSE	Mean ± *σ*	RMSE	Mean ± *σ*
MH01	0.14	0.12 ± 0.08	0.11	0.09 ± 0.05
MH02	0.11	0.10 ± 0.04	0.07	0.06 ± 0.04
F2K	0.14	0.12 ± 0.08	0.11	0.09 ± 0.05

**Table 2:** Execution time (in ms per frame) itemized in sequence *Machine Hall 01*.

		Intel(R) Core(TM) i5-3330		Intel Compute Stick	
Thread	Function	SDVL	SDVL-ORB	SDVL	SDVL-ORB
*Tracking*	Frame Initialization	4.73 ± 0.84	4.67 ± 0.77	9.00 ± 1.24	9.11 ± 1.31
	Image Alignment	1.95 ± 0.80	1.10 ± 0.45	9.61 ± 4.94	6.56 ± 2.33
	Feature Matching	2.95 ± 0.82	11.12 ± 4.29	7.47 ± 2.24	40.15 ± 12.51
	Pose update	0.54 ± 0.23	0.32 ± 0.20	1.41 ± 0.92	1.35 ± 2.14
	Total	10.14 ± 2.19	17.25 ± 5.19	28.27 ± 8.59	58.36 ± 15.34
*Mapping*	Update 3D Points	19.87 ± 8.26	13.63 ± 7.15	54.12 ± 18.69	44.75 ± 21.29
	Initialize 3D Points	10.90 ± 4.88	20.54 ± 10.00	39.89 ± 16.82	66.63 ± 29.16
	Bundle Adjustment	50.28 ± 19.21	23.39 ± 8.91	216.23 ± 72.26	139.21 ± 50.48
	Total with BA	88.47 ± 21.88	63.02 ± 19.75	311.86 ± 76.63	252.21 ± 77.61
	Total	34.69 ± 30.11	21.13 ± 19.32	108.08 ± 112.01	81.48 ± 87.96

**Table 3:** Accuracy comparative (in meters).

Sequence	SDVL	SDVL-ORB	PTAM	SVO	LSD-SLAM	ORB-SLAM	DSO	DSO-Fast
MH01	0.147	0.112	X	0.135	0.254	0.044	0.057	0.067
MH02	0.115	0.077	X	0.052	1.395	0.035	0.046	0.043
F1F	0.026	0.041	0.069 *	0.018 *	0.366	0.013	0.019	0.020
F2D	0.054	0.034	X	X	0.103	0.027	0.020	0.021
ZU	1.112	0.548	X	0.331	0.508	0.429	1.915	0.338

**Table 4:** Efficiency comparative (in milliseconds).

Sequence	SDVL	SDVL-ORB	PTAM	SVO	LSD-SLAM	ORB-SLAM	DSO	DSO-Fast
MH01	10.14	17.25	X	3.18	16.63	34.72	38.99	18.72
MH02	9.93	16.99	X	3.14	16.87	30.62	41.08	17.88
F1F	6.06	8.49	5.15 *	3.29 *	14.53	19.39	50.98	21.04
F2D	6.15	10.32	X	X	12.46	27.07	33.54	14.50
ZU	8.47	16.34	X	2.36	17.47	26.16	24.23	8.62

## References

[1] Dellaert F., Burgard W., Fox D., Thrun S. Using the condensation algorithm for robust, vision-based mobile robot localization. Proceedings of the IEEE Computer Society Conference on Computer Vision and Pattern Recognition.

[2] Davison A.J. Real-Time Simultaneous Localisation and Mapping with a Single Camera. Proceedings of the ICCV.

[3] Davison A.J., Reid I.D., Molton N.D., Stasse O. (2007). MonoSLAM: Real-time single camera SLAM. IEEE Trans. Pattern Anal. Mach. Intell..

[4] Klein G., Murray D. Parallel tracking and mapping for small AR workspaces. Proceedings of the 2007 6th IEEE and ACM International Symposium on Mixed and Augmented Reality ISMAR 2007.

[5] Strasdat H., Montiel J.M.M., Davison A.J. (2012). Visual SLAM: Why filter?. Image Vis. Comput..

[6] Rosten E., Drummond T. Machine learning for high-speed corner detection. Proceedings of the Computer Vision—ECCV 2006.

[7] Strasdat H., Davison A.J., Montiel J.M., Konolige K. Double window optimisation for constant time visual SLAM. Proceedings of the 2011 IEEE International Conference on Computer Vision (ICCV).

[8] Mur-Artal R., Montiel J.M.M., Tardos J.D. (2015). ORB-SLAM: A versatile and accurate monocular SLAM system. IEEE Trans. Robot..

[9] Rublee E., Rabaud V., Konolige K., Bradski G. ORB: An efficient alternative to SIFT or SURF. Proceedings of the 2011 IEEE International Conference on Computer Vision (ICCV).

[10] Newcombe R.A., Davison A.J. Live dense reconstruction with a single moving camera. Proceedings of the 2010 IEEE Conference on Computer Vision and Pattern Recognition (CVPR).

[11] Newcombe R.A., Lovegrove S.J., Davison A.J. DTAM: Dense tracking and mapping in real-time. Proceedings of the 2011 IEEE International Conference on Computer Vision (ICCV).

[12] Engel J., Schöps T., Cremers D. (2014). LSD-SLAM: Large-scale direct monocular SLAM. Lecture Notes in Computer Science, Proceedings of the European Conference on Computer Vision, Zurich, Switzerland, 6–12 Deptember 2014.

[13] Engel J., Koltun V., Cremers D. (2018). Direct sparse odometry. IEEE Trans. Pattern Anal. Mach. Intell..

[14] Gao X., Wang R., Demmel N., Cremers D. LDSO: Direct Sparse Odometry with Loop Closure. Proceedings of the International Conference on Intelligent Robots and Systems (IROS).

[15] Forster C., Pizzoli M., Scaramuzza D. SVO: Fast semi-direct monocular visual odometry. Proceedings of the 2014 IEEE International Conference on Robotics and Automation (ICRA).

[16] Bescos B., Fácil J.M., Civera J., Neira J. (2018). DynaSLAM: Tracking, Mapping and Inpainting in Dynamic Scenes. IEEE Robot. Autom. Lett..

[17] Labbe M., Michaud F. Online global loop closure detection for large-scale multi-session graph-based SLAM. Proceedings of the 2014 IEEE/RSJ International Conference on Intelligent Robots and Systems (IROS 2014).

[18] Florido A.M., Montero F.R., Plaza J.M.C. (2018). Robust 3D Visual Localization Based on RTABmaps. Advancements in Computer Vision and Image Processing.

[19] Vogiatzis G., Hernández C. (2011). Video-based, real-time multi-view stereo. Image Vis. Comput..

[20] Civera J., Davison A.J., Montiel J.M.M. (2008). Inverse depth parametrization for monocular SLAM. IEEE Trans. Robot..

[21] Baker S., Matthews I. (2004). Lucas-kanade 20 years on: A unifying framework. Int. J. Comput. Vis..

[22] Martin J., Crowley J.L. (1995). Comparison of correlation techniques. Intelligent Autonomous Systems.

[23] Civera J., Grasa O.G., Davison A.J., Montiel J.M.M. (2010). 1-Point RANSAC for extended Kalman filtering: Application to real-time structure from motion and visual odometry. J. Field Robot..

[24] Fischler M.A., Bolles R.C. (1981). Random sample consensus: A paradigm for model fitting with applications to image analysis and automated cartography. Commun. ACM.

[25] Nistér D. (2004). An efficient solution to the five-point relative pose problem. IEEE Trans. Pattern Anal. Mach. Intell..

[26] Lucas B.D., Kanade T. An iterative image registration technique with an application to stereo vision. Proceedings of the 7th International Joint Conference on Artificial Intelligence.

[27] Shi J. Good features to track. Proceedings of the 1994 IEEE Computer Society Conference on Computer Vision and Pattern Recognition CVPR’94.

[28] Triggs B., McLauchlan P.F., Hartley R.I., Fitzgibbon A.W. (1999). Bundle adjustment—A modern synthesis. Lecture Notes in Computer Science, Proceedings of the International Workshop on Vision Algorithms, Corfu, Greece, 20–25 September 1999.

[29] Sturm J., Engelhard N., Endres F., Burgard W., Cremers D. A benchmark for the evaluation of RGB-D SLAM systems. Proceedings of the 2012 IEEE/RSJ International Conference on Intelligent Robots and Systems (IROS).

[30] Burri M., Nikolic J., Gohl P., Schneider T., Rehder J., Omari S., Achtelik M.W., Siegwart R. (2016). The EuRoC micro aerial vehicle datasets. Int. J. Robot. Res..

[31] Majdik A.L., Till C., Scaramuzza D. (2017). The Zurich urban micro aerial vehicle dataset. Int. J. Robot. Res..

[32] PTAM Original Source Code. http://www.robots.ox.ac.uk/~gk/PTAM.

[33] SVO Original Source Code Repository. https://github.com/uzh-rpg/rpg_svo.

[34] LSD-SLAM Original Source Code Repository. https://github.com/tum-vision/lsd_slam.

[35] ORB-SLAM Original Source Code Repository. https://github.com/raulmur/ORB_SLAM2.

[36] DSO Original Source Code Repository. https://github.com/JakobEngel/dso.

